# Effects of dexrazoxane on doxorubicin-related cardiotoxicity and second malignant neoplasms in children with osteosarcoma: a report from the Children’s Oncology Group

**DOI:** 10.1186/s40959-019-0050-9

**Published:** 2019-10-28

**Authors:** Lisa M. Kopp, Richard B. Womer, Cindy L. Schwartz, David H. Ebb, Vivian I. Franco, David Hall, Donald A. Barkauskas, Mark D. Krailo, Holcombe E. Grier, Paul A. Meyers, Leonard H. Wexler, Neyssa M. Marina, Katherine A. Janeway, Richard Gorlick, Mark L. Bernstein, Steven E. Lipshultz, for the Children’s Oncology Group

**Affiliations:** 1 Department of Epidemiology and Biostatistics, Mel and Enid Zuckerman College of Public Health, The University of Arizona, 1295 N Martin Ave. PO Box 245210, Tucson, AZ 85724 USA; 2 University of Arizona Cancer Center, University of Arizona, Tucson, AZ USA; 3 Children’s Hospital of Philadelphia, Philadelphia, PA USA; 4 Children’s Hospital of Wisconsin, Medical College of Wisconsin, Milwaukee, WI USA; 5 Department of Pediatric Hematology-Oncology, Massachusetts General Hospital, Boston, MA USA; 6 Department of Pediatrics, University at Buffalo, Oishei Children’s Hospital, Roswell Park Comprehensive Cancer Center, Buffalo, NY USA; 7 Children’s Oncology Group, Monrovia, CA USA; 8 Department of Preventive Medicine, Keck School of Medicine, University of Southern California, Los Angeles, CA USA; 9 Dana-Farber Cancer Institute, Boston, MA USA; 10 Memorial Sloan Kettering Cancer Center, New York, NY USA; 11 Five Prime Therapeutics, Inc., South San Francisco, CA USA; 12 MD Anderson Cancer Center, Houston, TX USA; 13 IWK Health Centre, Halifax, Nova Scotia Canada

**Keywords:** Osteosarcoma, Pediatrics, Cardiotoxicity, Doxorubicin

## Abstract

**Background:**

Dexrazoxane protects from lower-cumulative-dose doxorubicin cardiotoxicity, but the effect of dexrazoxane in children with sarcoma treated with higher-cumulative-dose doxorubicin is unknown.

**Methods:**

We evaluated children with osteosarcoma (OS) on two Children’s Oncology Group trials with higher dose doxorubicin (375–600 mg/m^2^) preceded by dexrazoxane (10:1 dexrazoxane:doxorubicin dosing). They were evaluated after the minimum expected treatment time (METT), defined as 28 weeks. Cardiotoxicity was identified by echocardiography and serum N-terminal pro-brain natriuretic peptide (NT-proBNP). Second malignant neoplasm (SMN) data was collected.

**Results:**

All children had normal left ventricular (LV) systolic function as measured by LV fractional shortening and no heart failure. The end-diastolic septal thickness *Z-*scores (*P* < 0.01) and LV mass *Z-*scores (*P* < 0.01) were significantly smaller than normal for body-surface area in both sexes. The average LV mass *Z-*scores were significantly smaller for girls (*P* < 0.01) and marginally smaller for boys (*P* = 0.06). Girls had significantly smaller LV end-diastolic dimension *Z-*scores normalized to BSA (*P* < 0.01) compared to healthy controls and had significant increases in NT-proBNP. Four children developed SMNs as first events, a rate similar to historical controls.

**Conclusions:**

Dexrazoxane prevented LV dysfunction and heart failure in children with OS receiving higher dose doxorubicin. However, LV structural changes were not fully prevented, especially in girls. As a result, hearts become abnormally small for body size, resulting in higher LV stress. Dexrazoxane did not increase the risk of SMN. Dexrazoxane should be used in this population, particularly for girls, to mitigate anthracycline-induced cardiotoxicity.

**Trial registrations:**

ClinicalTrials.gov: NCT00003937 (P9754) registered 1 Nov 1999, and NCT00023998 (AOST0121) registered 13 Sept 2001.

## Background

Osteosarcoma (OS) is the most common malignant bone tumor in children and adolescents. Multi-agent chemotherapy and surgery have greatly improved survival for patients with localized disease [1, 2]. Doxorubicin is perhaps the most important agent for the treatment of OS [3]. Most protocols use cumulative doses of 375–450 mg/m^2^, which can be associated with cardiotoxicity during treatment, as well as long-term cardiovascular morbidity and early mortality [4, 5].

The Childhood Cancer Survivor Study (CCSS) reported cardiac death as the second most common cause of late mortality in childhood cancer survivors, particularly in those receiving high doses of anthracyclines [6, 7]. The hazard ratio of adverse cardiac outcomes in survivors who received ≥250 mg/m^2^ of an anthracycline was up to fivefold higher than in those receiving less anthracycline [6, 7]. These data rely on self-reported events and may not reflect the full spectrum of cardiac injury. While dexrazoxane mitigates anthracyclines’ cardiotoxic effects [8, 9], reports of an increased risk of second malignant neoplasm (SMN) have limited its use in pediatric oncology [10, 11].

Two Children’s Oncology Group (COG) trials for OS provide a large cohort for assessing the impact of dexrazoxane in children and adolescents treated with higher cumulative doses of doxorubicin [12, 13]. We have previously published data from these trials on left ventricular (LV) systolic function (fractional shortening) and LV stress (N-terminal pro-brain natriuretic peptide, NT-proBNP) [12, 13]. However, we have also noted that late anthracycline-associated cardiotoxicity in long-term survivors of childhood ALL and OS results from two distinct pathways [14–18].

The first pathway is a direct effect of doxorubicin. Doxorubicin promotes formation of free radicals that disrupt cardiomyocyte cellular constituents, particularly in the mitochondria. This impairs the intrinsic health of the remaining cardiomyocytes and depresses contractility, while also causing cell death [8, 19].

The second pathway is the development of a restrictive cardiomyopathy due to LV dimensions and wall thickness that are abnormally small for body-surface area, resulting in increased LV afterload (stress) that may ultimately reduce LV function and lead to heart failure.

The objectives of the current analysis were to determine: 1) the incidence of cardiotoxicity in children and adolescents with OS receiving high cumulative dose doxorubicin (450–600 mg/m^2^) or trastuzumab plus dexrazoxane in the COG OS clinical trials P9754 and AOST0121 [12, 13], 2) the cumulative incidence of SMN.

## Methods

### Treatment protocols

Children, adolescents, and young adults with measurable, newly diagnosed, high-grade OS were enrolled on one of two multi-institutional COG OS protocols: P9754 between 1999 and 2002 [12, 13], and on AOST0121 between 2001 and 2005. Written informed consent was obtained from children and/or their guardians according to local institutional review board guidelines before starting therapy.

P9754 [13] included three sequential phase II pilot studies with a complex design for localized OS. Treatment was based on MAP (methotrexate, doxorubicin, cisplatin), with or without added ifosfamide, ifosfamide-etoposide (IE), or intensification of doxorubicin (cumulative dose 600 mg/m^2^) in patients whose tumor showed a poor histologic response. AOST0121 [12] was a phase II study for metastatic OS; patients whose tumors had overexpression of human epidermal growth factor receptor 2 (HER2) received MAPIE with trastuzumab, while the other patients received MAP.

Both studies required normal LV function (LV fractional shortening of ≥28% or LV ejection fraction ≥50%) at enrollment. Patients with a history of pericarditis, myocarditis, and symptomatic dysrhythmias or conduction disturbances were excluded.

### Therapy

All patients received MAP with dexrazoxane given in a 10:1 ratio before each dose of doxorubicin. Surgical resection of primary and metastatic sites was planned after week 10 of chemotherapy in both studies. Echocardiograms were to be obtained at baseline, at specified intervals during therapy, and after completing treatment. Blood was to be collected at baseline, before and after doxorubicin administration (30 h after doses 3, 5, 6, 7, and 8 on P9754 and 24 to 72 h after doses 3 and 5 on AOST0121), and at the end of treatment.

### Echocardiographic and biomarker measurements

Cardiotoxicity was defined by echocardiographic criteria and by serum concentrations of cardiac troponin-T (cTnT), which measures injury to cardiomyocytes, and of N-terminal pro-brain natriuretic peptide (NT-proBNP), which is elevated in cardiomyopathy associated with increased myocardial stress from ventricular pressure or volume overload [20–23]. Central re-analyses of the original echocardiogram tracings from both studies were performed using computer digital analysis with methods used previously to establish normative data in children [24]. Echocardiograms were only included in this analysis if they were centrally reviewed and the patient had completed therapy.

Serum cTnT levels were determined at a central laboratory using the Elecsys Troponin-T STAT Immunoassay (Roche Diagnostics Corporation; sensitivity, 0.01 ng/mL); any detectable amount was considered abnormal. Levels of NT-proBNP were measured by using the Elecsys immunoanalyzer (Roche Diagnostics; sensitivity, 5 pg/mL); the cardiomyopathy risk threshold was defined as ≥100 pg/mL, and the risk threshold for heart failure was defined as ≥400 pg/mL [25, 26]. Central investigators evaluating echocardiograms and cardiac biomarkers were blinded to the patients’ clinical status and treatment assignments but were aware of the study objectives. Results were not reported to the care centers.

### Second malignant neoplasms

SMN were identified through National Cancer Institute-mandated adverse event reporting. The cumulative doses of drugs given that have been associated with increased SMN risk are shown in Table 1.

**Table 1:** SMN^a^ Risk

Treatment Arm	Dexrazoxane	Doxorubicin	Ifosfamide	Etoposide
AOST0121 HER2+	3.75 g/m^2^	375 mg/m^2^	51 g/m^2^	1.5 g/m^2^
AOST0121 HER2-	3.75 g/m^2^	375 mg/m^2^	51 g/m^2^	1.5 g/m^2^
P9754 Pilot 1 Good Response	4.5 g/m^2^	450 mg/m^2^		
P9754 Pilot 1 Standard Response	6 g/m^2^	600 mg/m^2^		
P9754 Pilot 2 Good Response	4.5 g/m^2^	450 mg/m^2^	27 g/m^2^	
P9754 Pilot 2 Standard Response	6 g/m^2^	600 mg/m^2^	45 g/m^2^	
P9754 Pilot 3 Good Response	4.5 g/m^2^	450 mg/m^2^	27 g/m^2^	
P9754 Pilot 3 Standard Response	4.5 g/m^2^	450 mg/m^2^	60 g/m^2^	1.5 g/m^2^

^a^*SMN* second malignant neoplasms

### Statistical methods

The Proc Mixed Procedure in SAS 9.4 was used for all regression models with a compound symmetry correlation structure for repeated measures within patients. The NT-proBNP raw data was log-transformed for analysis.

To adjust for growth-related changes, echocardiographic data were standardized by age (LV fractional shortening) or body-surface area (BSA; LV mass, LV dimension, septal thickness, and LV wall thickness). We calculated a *Z-*score of deviation from normal values based on data from 285 normal children and adolescents [25] in whom BSA ranged from 0.2 to 2.2 m^2^, who had normal height, weight, height-for-weight percentiles, and blood pressure; and had no evidence of cardiac or other disorders.

Since the times of echocardiographic and NT-proBNP measurements varied among protocols and patients, we defined a minimum expected treatment time (METT) of 28 weeks from enrollment. We then partitioned the follow-up assessment points equally according the interval from METT: 0–37 days, 38–81 days, and > 81 days. No echocardiogram performed after disease progression or SMN was considered in the analysis.

Time to SMN was the number of days from enrollment to diagnosis of an SMN unless disease progression or death occurred first as a competing event. Patients not experiencing an event were considered censored at last contact. The cumulative incidence of SMNs was calculated using the methods of Gray [27]. The cumulative incidence of SMNs in the OS study, INT-0133 [1], which did not include dexrazoxane, was the basis of the historical comparison.

## Results

### Patients

A total of 316 patients from P9754 and AOST0121 were available for evaluation, with a male:female ratio of 1.2:1 and a mean age at enrollment of 13.7 years as seen in Table 2. The mean and median follow-up time was 73 months (77 months for P9754 and 53 months for AOST0121) and 80 months (82 months for P9754 and 55 months for AOST1521) respectively. Echocardiograms were available for central review from 81 patients; 68 had cTnT data available and 59 had NT-proBNP data available as shown in Table 3. There was a substantial amount of data missing from patients enrolled on both studies as shown in Table 4.

**Table 2:** Patient Characteristics

Study and Treatment Arm		Mean Age at Enrollment^a^	Patient Sex^b^		Total	Expected Weeks	Dex^c^	Doxorubicin	Trastuzumab
			Male	Female					
AOST0121	AOST0121 HER2 Negative	15.1 (3.6)	33 (60.0%)	22 (40.0%)	55 (100%)	34	3.75 g/m^2^	375 mg/m^2^	70 mg/kg
	AOST0121 HER2 Positive	13.7 (2.7)	19 (47.5%)	21 (52.5%)	40 (100%)	34	3.75 g/m^2^	375 mg/m^2^	
P9754	P9754 Pilot 1 Good Response	13.5 (5.0)	16 (47.1%)	18 (52.9%)	34 (100%)	28	4.5 g/m^2^	450 mg/m^2^	
	P9754 Pilot 1 Standard Response	13.9 (4.4)	48 (62.3%)	29 (37.7%)	77 (100%)	29	6 g/m^2^	600 mg/m^2^	
	P9754 Pilot 2 Good Response	13.9 (3.9)	7 (33.3%)	14 (66.7%)	21 (100%)	28	4.5 g/m^2^	450 mg/m^2^	
	P9754 Pilot 2 Standard Response	13.8 (4.5)	20 (60.6%)	13 (39.4%)	33 (100%)	31	6 g/m^2^	600 mg/m^2^	
	P9754 Pilot 3 Good Response	14.3 (4.0)	12 (63.2%)	7 (36.8%)	19 (100%)	28	4.5 g/m^2^	450 mg/m^2^	
	P9754 Pilot 3 Standard Response	13.4 (4.6)	20 (54.1%)	17 (45.9%)	37 (100%)	36	4.5 g/m^2^	450 mg/m^2^	

^a^Mean (SD), years

^b^Number (%)

^c^Dex, Dexrazoxane

**Table 3:** Number of Patients with Data Available After METT^a^ (28 weeks)

Variable	LV Fractional Shortening *Z-*scores	LV End-Diastolic Dimension *Z-*scores	LV End-Diastolic Posterior Wall Thickness *Z-*scores	LV Thickness to Dimension Ratio *Z-*scores	End-Diastolic Septal Thickness *Z-*scores	LV Mass *Z-*scores	NT-proBNP
Overall	*n* = 81	*n* = 78	*n* = 78	*n* = 82	*n* = 70	*n* = 74	*n* = 59
AOST0121	*n* = 8	*n* = 5	*n* = 5	*n* = 9	*n* = 5	*n* = 5	*n* = 15
P9754	*n* = 73	*n* = 73	*n* = 73	*n* = 73	*n* = 65	*n* = 69	*n* = 44

^a^*METT* minimum expected treatment time

**Table 4:** Number of Patients with Data Available and Number Excluded due to Missing Data^a^ After METT^b^

Variable	LV Fractional Shortening *Z-*scores	LV End-Diastolic Dimension *Z-*scores	LV End-Diastolic Posterior Wall Thickness *Z-*scores	LV Thickness to Dimension Ratio *Z-*scores	End-Diastolic Septal Thickness *Z-*scores	LV Mass *Z-*scores	NT-proBNP
Overall	*n* = 229	*n* = 224	*n* = 221	*n* = 228	*n* = 212	*n* = 214	*n* = 156
	(*n* = 148)	(*n* = 146)	(*n* = 143)	(*n* = 146)	(*n* = 142)	(*n* = 140)	(*n* = 97)
AOST0121	*n* = 90	*n* = 85	*n* = 83	*n* = 89	*n* = 83	*n* = 82	*n* = 42
	(*n* = 82)	(*n* = 80)	(*n* = 78)	(*n* = 80)	(n = 78)	(*n* = 77)	(*n* = 27)
P9754	*n* = 139	*n* = 139	*n* = 138	*n* = 139	*n* = 129	*n* = 132	*n* = 114
	(*n* = 66)	(n = 66)	(*n* = 65)	(n = 66)	(*n* = 64)	(*n* = 63)	(*n* = 70)

^a^Number of patients with missing data in parenthesis for each variable

^b^*METT* minimum expected treatment time

### Echocardiographic measurements

**Fig. 1:**
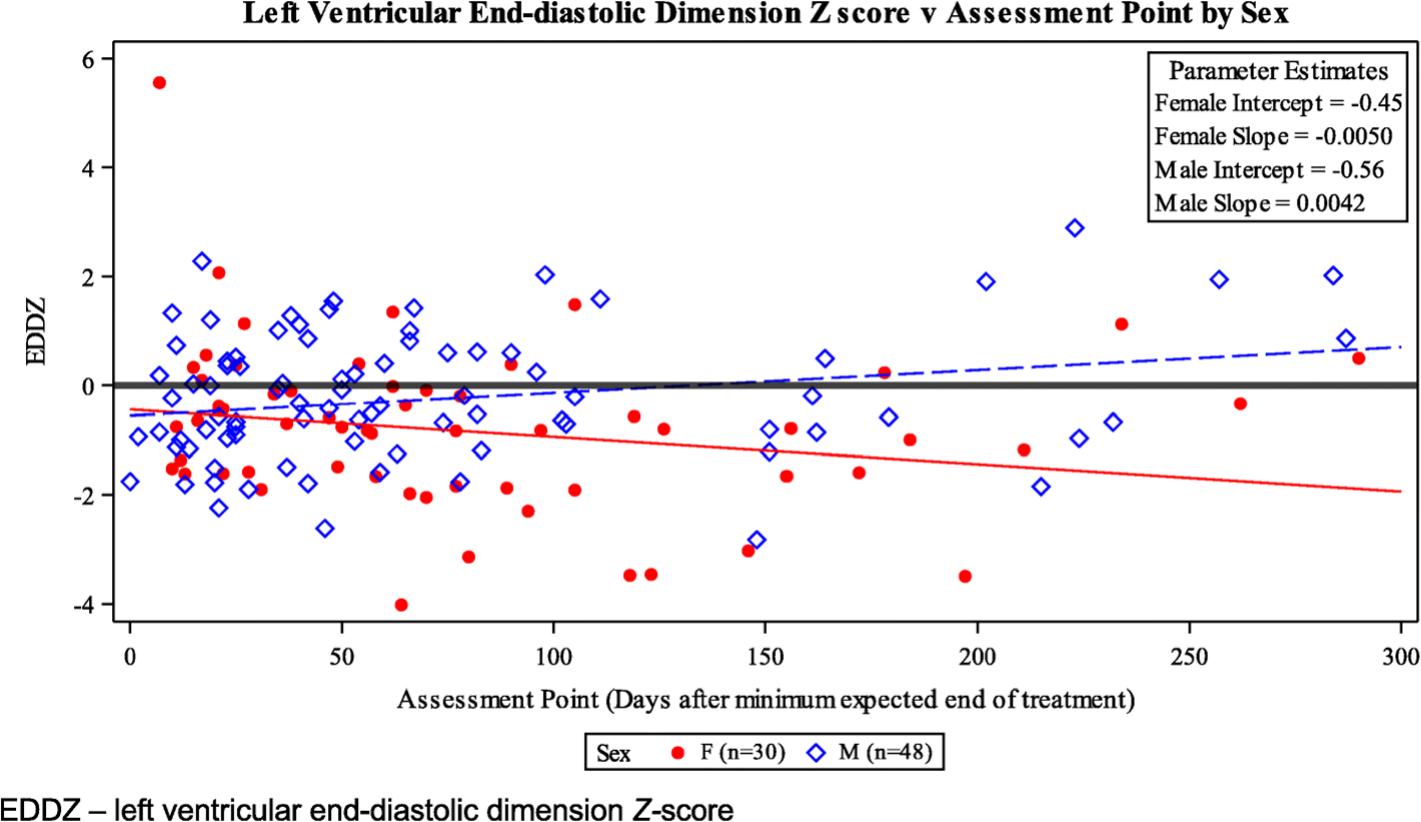
Changes in Left Ventricular End-Diastolic Dimension *Z-*scores in Patients, by Sex

We found no predictors of LV fractional shortening, LV end-diastolic posterior wall thickness, or LV thickness-to-dimension ratio *Z-*scores (a marker of pathologic adverse LV remodeling) for patients evaluated after the METT. However, the LV end-diastolic septal thickness *Z*-scores was significantly smaller than normal for BSA in both sexes (*P* < 0.01; Table 5) while the LV wall thickness and LV mass *Z*-scores were significantly smaller than normal for girls (*P* < 0.01; Table 5) and marginally smaller for boys but not statistically significant (*P* = 0.06; Table 5). A significant association between the assessment point (the follow-up points divided equally according to the interval from METT: 0–37 days, 38–81 days, and > 81 days) and the patient’s sex was found (*P* < 0.01; Table 6), with LV end-diastolic dimension *Z-*scores decreasing over time in girls and increasing over time in boys (Fig. 1).

**Table 5:** Echocardiographic and NT-proBNP measurements of Patients Evaluated After METT^a^ (28 weeks)

Measurement	Group	N	Mean	95% Confidence Interval for Mean	*P* ^b^
NT-proBNP^2^	All Patients	59	47.3^c^	(36.3, 61.6)^c^	< 0.01
LV End-Diastolic Posterior Wall Thickness Z-score	All Patients	78	−0.29	(− 0.60, 0.02)	0.06
	Female	30	− 0.57	(−1.04, − 0.09)	0.02
	Male	48	−0.10	(− 0.50, 0.30)	0.63
End-Diastolic Septal Thickness Z-score	All Patients	70	−0.84	(−1.2, − 0.48)	< 0.01
	Female	27	−1.20	(−1.76, −0.64)	< 0.01
	Male	43	−0.60	(−1.06, − 0.15)	0.01
LV Fractional Shortening *Z-*score	All Patients	81	−0.19	(−0.54, 0.17)	0.30
	Female	32	−0.46	(−1.02, 0.10)	0.10
	Male	49	0.00	(−0.46, 0.47)	0.99
LV Mass Z-score	All Patients	74	−0.74	(−1.06, − 0.42)	< 0.01
	Female	29	−1.23	(−1.71, −0.75)	< 0.01
	Male	45	−0.39	(−0.80, 0.01)	0.06

^a^*METT* minimal expected treatment time

^b^In the Z-score columns, a Z-score of 0 represents the average value for a healthy child of the same age. Each *P*-value for the Z-score measurements is from the two-sided hypothesis test with the null hypothesis H_0_: the population mean for the Z-scores is 0. (A Z-score of 0 represents the average value for a healthy child of the same age.) The *P*-value for NT-proBNP is from the two-sided hypothesis test with null hypothesis H_0_: the population geometric mean is 100

^**c**^NT-proBNP is a raw score. Analysis for the NT-proBNP was done using a natural log transformation to stabilize the variance. The back-transformed NT-proBNP numbers are presented here for ease of interpretability. Due to the back transformations, these represent geometric means

**Table 6:** Cardiac and NT-proBNP Measurements

Prediction Factor, Model	*P* ^*a*^
Left Ventricular End-Diastolic Dimension Z-Scores	
Univariate Model	
Sex	0.04
Final Model	
Assessment Point^b^	0.77
Sex^2^	0.74
Assessment Point* Sex Interaction Term	< 0.01
Male Slope^c^	0.02
Female Slope^c^	0.02
NT pro-BNP, Both Studies Combined	
Sex	0.02
NT pro-BNP, P9754	
Univariate Models	
Assessment Point	0.02
Sex	0.02
Final Model	
Assessment Point	< 0.01
Sex	< 0.01
Assessment Point * Sex Interaction Term^d^	0.59
NT pro-BNP, AOST0121^e^	
Final Model	
Assessment Point^f^	0.05
Sex	0.03
Assessment Point * Sex Interaction Term	0.03
Male Slope^c^	0.03
Female Slope^c,f^	0.08

^a^All *P*-value are Type 3 *p*-values from models produced by Proc Mixed in SAS 9.4, unless otherwise specified

^b^The main effects in the final model were not significant, but were kept because their interaction term was significant

^c^*P*-value obtained by a linear hypothesis H0: Slope = 0 using Proc Mixed in SAS 9.4

^d^The interaction term for assessment point and sex was not found to be significant and was dropped from the final model

^e^Neither assessment point nor sex were significant in their respective univariate models

^f^Marginally significant

### Cardiac biomarker assays

None of the 68 children with biomarker data in either trial had detectable cTnT concentrations at any time.

Overall the values for NT-proBNP were below the level of concern for heart failure (less than 400 pg/mL) for the 59 children and adolescents evaluated after the minimum expected treatment time (*P* < 0.01; Table 5). However, NT-proBNP concentrations were significantly associated with study (AOST0121 and P9754) (*P* = 0.02; Table 6). Because of this association, further analyses were done separately for P9754 and AOST0121 (Table 6).

**Fig. 2:**
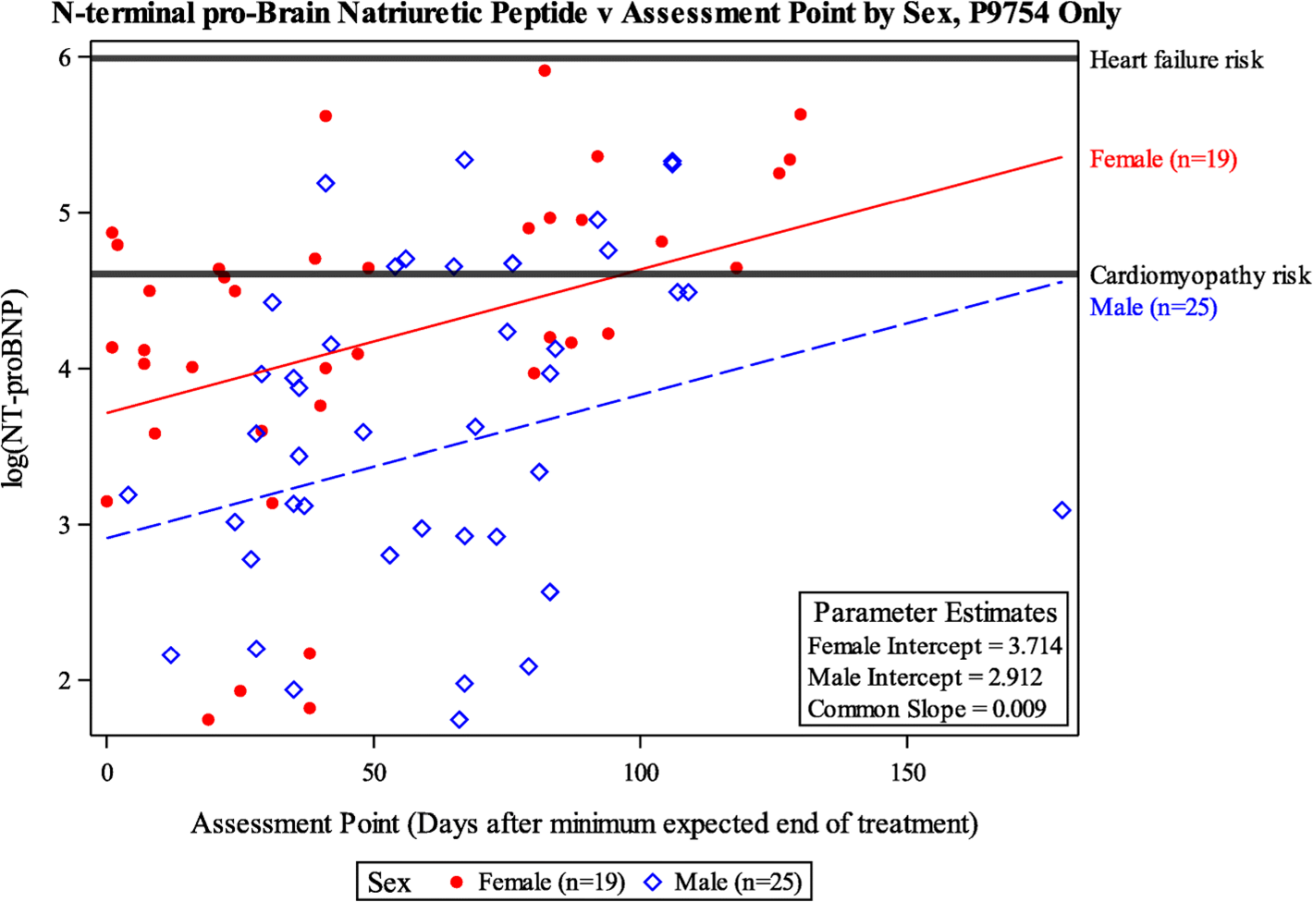
Risk of Cardiomyopathy and Heart Failure, by NT-proBNP, among Patients Enrolled on P9754, by Time Since Completing Treatment and Sex. Cardiomyopathy and heart failure risk thresholds for this NT-proBNP assay in children with cardiomyopathy have been determined to be ≥100 pg/mL and ≥ 400 pg/mL, respectively [25, 26]. The y-axis of this figure shows the log transformed values of NT-proBNP. The horizontal lines indicating cardiomyopathy and heart failure thresholds correspond to 100 pg/mL and 400 pg/mL, respectively before they were log transformed

In P9754, the values of log [NT-proBNP] increased over time for both sexes (*P* < 0.01) and were significantly higher in girls than in boys (*P* < 0.01) (Table 6). However, there was no significant interaction between assessment point and sex (*P* = 0.59; Table 6), represented by the common slope in Fig. 2. No P9754 patients had log [NT-proBNP] measurements which were in the range of heart failure risk (Fig. 2).

**Fig. 3:**
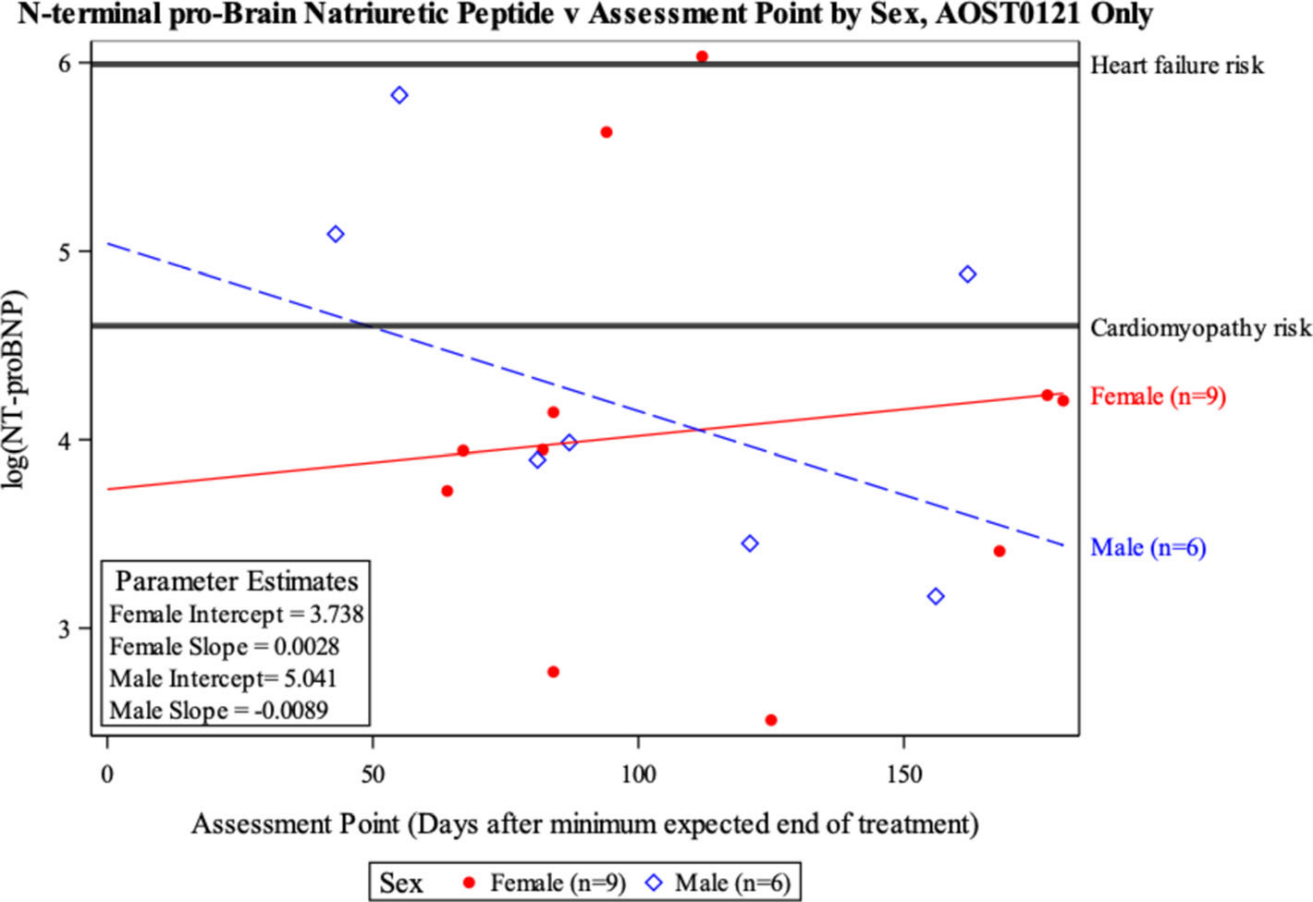
Risk of Cardiomyopathy and Heart Failure, as Assessed by by NT-proBNP, among Patients enrolled on the COG AOST0121 Study, by Sex.. Cardiomyopathy was defined by having a NT-proBNP ≥100 pg/mL and risk of heart failure was defined as an NT-proBNP ≥400 pg/mL [25, 26]. The y-axis of this figure shows the log [NT-proBNP]. The horizontal lines indicating cardiomyopathy and heart failure thresholds correspond to 100 pg/mL and 400 pg/mL, respectively before they were log transformed

For AOST0121, patient sex (*P* = 0.03), assessment point (*P* = 0.05), and the interaction term between sex and assessment point (*P* = 0.03) were all found to be significant in the final model for log [NT-proBNP] (Table 6). The final model showed boys’ log [NT-proBNP] decreasing over time (*P* = 0.03) and girls’ log [NT-proBNP] increasing over time (*P* = 0.08) (Table 6). One girl on AOST0121 had a log [NT-proBNP] value in the range of heart failure risk (Figure 3).

For a complete view of additional cardiac data, Table 7 reports all collected echocardiographic *Z*-scores and NT-proBNP measurements after METT.

**Table 7:** Echocardiographic and NT-proBNP of Patients After METT^a^ (28 weeks)^b^

Variable	LV Fractional Shortening *Z-*scores	LV End-Diastolic Dimension *Z-*scores	LV End-Diastolic Posterior Wall Thickness *Z-*scores	LV Thickness to Dimension Ratio *Z-*scores	End-Diastolic Septal Thickness *Z-*scores	LV Mass *Z-*scores	NT-proBNP^c^
Overall	n = 81	n = 78	n = 78	n = 82	n = 70	n = 74	n = 59
	$\overline{x}$ = − 0.19	$\overline{x}$ = − 0.49	$\overline{x}$ = − 0.29	$\overline{x}$ = − 0.23	$\overline{x}$ = − 0.84	$\overline{x}$ = − 0.74	GM = 47.3
	(− 0.54, 0.17)	(− 0.76, − 0.21)	(− 0.60, 0.02)	(− 0.53, 0.07)	(− 1.2, − 0.48)	(− 1.06, − 0.42)	(36.3, 61.6)
	*P* = 0.30	*P* < 0.01	*P* = 0.06	*P* = 0.13	*P* < 0.01	*P* < 0.01	*P* < 0.01
Sex							
Female	n = 32	n = 30	*n* = 30	*n* = 33	n = 27	*n* = 29	*n* = 28
	$\overline{x}$ = − 0.46	$\overline{x}$ = − 0.82	$\overline{x}$ = − 0.57	$\overline{x}$ = − 0.29	$\overline{x}$ = − 1.20	$\overline{x}$ = − 1.23	GM = 63.2
	(− 1.02, 0.10)	(−1.24, − 0.40)	(− 1.04, − 0.09)	(− 0.75, 0.16)	(−1.76, − 0.64)	(− 1.71, − 0.75)	(43.8, 91.4)
	*P* = 0.10	*P* < 0.01	*P* = 0.02	*P* = 0.20	*P* < 0.01	*P* < 0.01	*P* = 0.02
Male	*n* = 49	*n* = 48	n = 48	n = 49	*n* = 43	n = 45	*n* = 31
	$\overline{x}$ = 0.00	$\overline{x}$ = − 0.26	$\overline{x}$ = − 0.10	$\overline{x}$ = − 0.18	$\overline{x}$ = − 0.60	$\overline{x}$ = − 0.39	GM = 36.1
	(− 0.46, 0.47)	(− 0.60, 0.08)	(− 0.50, 0.30)	(− 0.57, 0.21)	(− 1.06, − 0.15)	(−0.80, 0.01)	(25.3, 51.6)
	*P* = 0.99	*P* = 0.14	*P* = 0.63	*P* = 0.36	*P* = 0.01	*P* = 0.06	*P* < 0.01
Study							
AOST0121	n = 8	n = 5	n = 5	*n* = 9	n = 5	n = 5	n = 15
	$\overline{x}$ = − 0.70	$\overline{x}$ = − 0.61	$\overline{x}$ = − 0.64	$\overline{x}$ = 0.10	$\overline{x}$ = −3.82	$\overline{x}$ = − 0.86	GM = 60.5
	(−2.06, 0.67)	(−1.82, 0.59)	(− 2.06, 0.78)	(−0.97, 1.17)	(−5.05, − 2.59)	(−2.23, 0.50)	(35.0, 104.6)
	*P* = 0.31	*P* = 0.31	*P* = 0.37	*P* = 0.85	*P* < 0.01	*P* = 0.21	*P* = 0.07
P9754	*n* = 73	*n* = 73	n = 73	n = 73	n = 65	*n* = 69	*n* = 44
	$\overline{x}$ = −0.15	$\overline{x}$ = − 0.48	$\overline{x}$ = − 0.27	$\overline{x}$ = − 0.26	$\overline{x}$ = − 0.63	$\overline{x}$ = − 0.73	GM = 43.9
	(−0.52, 0.22)	(−0.76, − 0.20)	(−0.59, 0.04)	(− 0.57, 0.05)	(− 0.96, − 0.31)	(− 1.07, − 0.40)	(32.4, 59.3)
	*P* = 0.43	*P* < 0.01	*P* = 0.09	*P* = 0.10	*P* < 0.01	*P* < 0.01	*P* < 0.01
Assessment Point^d^							
0 to 37 days	n = 48	n = 48	*n* = 45	n = 45	*n* = 40	*n* = 38	*n* = 30
	$\overline{x}$ = −0.18	$\overline{x}$ = −0.38	$\overline{x}$ = − 0.40	$\overline{x}$ = − 0.43	$\overline{x}$ = − 0.39	$\overline{x}$ = − 0.67	GM = 32.6
	(−0.72, 0.35)	(−0.75, − 0.01)	(−0.85, 0.05)	(− 0.90, 0.03)	(− 0.80, 0.03)	(− 1.13, − 0.20)	(18.4, 57.7)
	*P* = 0.49	*P* = 0.05	*P* = 0.08	*P* = 0.06	*P* = 0.07	*P* < 0.01	*P* = 0.01
38 to 81 days	*n* = 42	n = 40	n = 38	*n* = 41	*n* = 36	*n* = 32	n = 32
	$\overline{x}$ = −0.13	$\overline{x}$ = −0.60	$\overline{x}$ = 0.15	$\overline{x}$ = 0.15	$\overline{x}$ = −0.82	$\overline{x}$ = −0.43	GM = 45.2
	(−0.70, 0.43)	(−1.00, − 0.21)	(−0.33, 0.64)	(− 0.33, 0.63)	(− 1.24, − 0.40)	(−0.93, 0.07)	(26.4, 77.3)
	*P* = 0.64	*P* < 0.01	*P* = 0.53	*P* = 0.53	*P* < 0.01	*P* = 0.09	*P* = 0.01
> 81 days	*n* = 39	*n* = 37	n = 37	n = 40	n = 29	n = 36	*n* = 34
	$\overline{x}$ = −0.24	$\overline{x}$ = −0.51	$\overline{x}$ = − 0.57	$\overline{x}$ = − 0.35	$\overline{x}$ = − 1.46	$\overline{x}$ = − 1.06	GM = 67.6
	(−0.82, 0.34)	(−0.91, − 0.10)	(− 1.05, − 0.09)	(−0.83, 0.12)	(− 1.91, − 1.02)	(−1.53, − 0.60)	(39.9, 114.5)
	*P* = 0.41	*P* = 0.01	*P* = 0.02	*P* = 0.14	*P* < 0.01	*P* < 0.01	*P* = 0.11

^a^*METT* minimal expected treatment time

^b^In the Z-score columns, a Z-score of 0 represents the average value for a healthy child of the same age

^**c**^NT-proBNP is a raw score. Analysis for the NT-proBNP was done using a natural log transformation to better adhere to the normality assumption for regression. The back-transformed NT-proBNP numbers are presented here for ease of interpretability. Due to the back transformations, these represent geometric means (GM). The *P*-value is from the two-sided hypothesis test with null hypothesis H_0_: the population GM is 100

^d^Assessment Point is the measure in days after the minimum expected treatment (28 weeks/196 days) and broken up into tertiles based on the 33.3rd and 66.7th percentiles of the assessment points of all measurements. Due to multiple measurements per patient, it is possible for patients to be in multiple tertiles

### Second malignant neoplasms

In AOST0121, 95 children and adolescents were included in the analysis and 6 patients were excluded: 1 was misdiagnosed for HER2 status and assigned the wrong treatment, 4 were ineligible for AOST0121, and 1 was excluded for both reasons. For P9754, 221 children and adolescents were included in the analysis, and 32 were excluded because they were declared ineligible during the study.

**Fig. 4:**
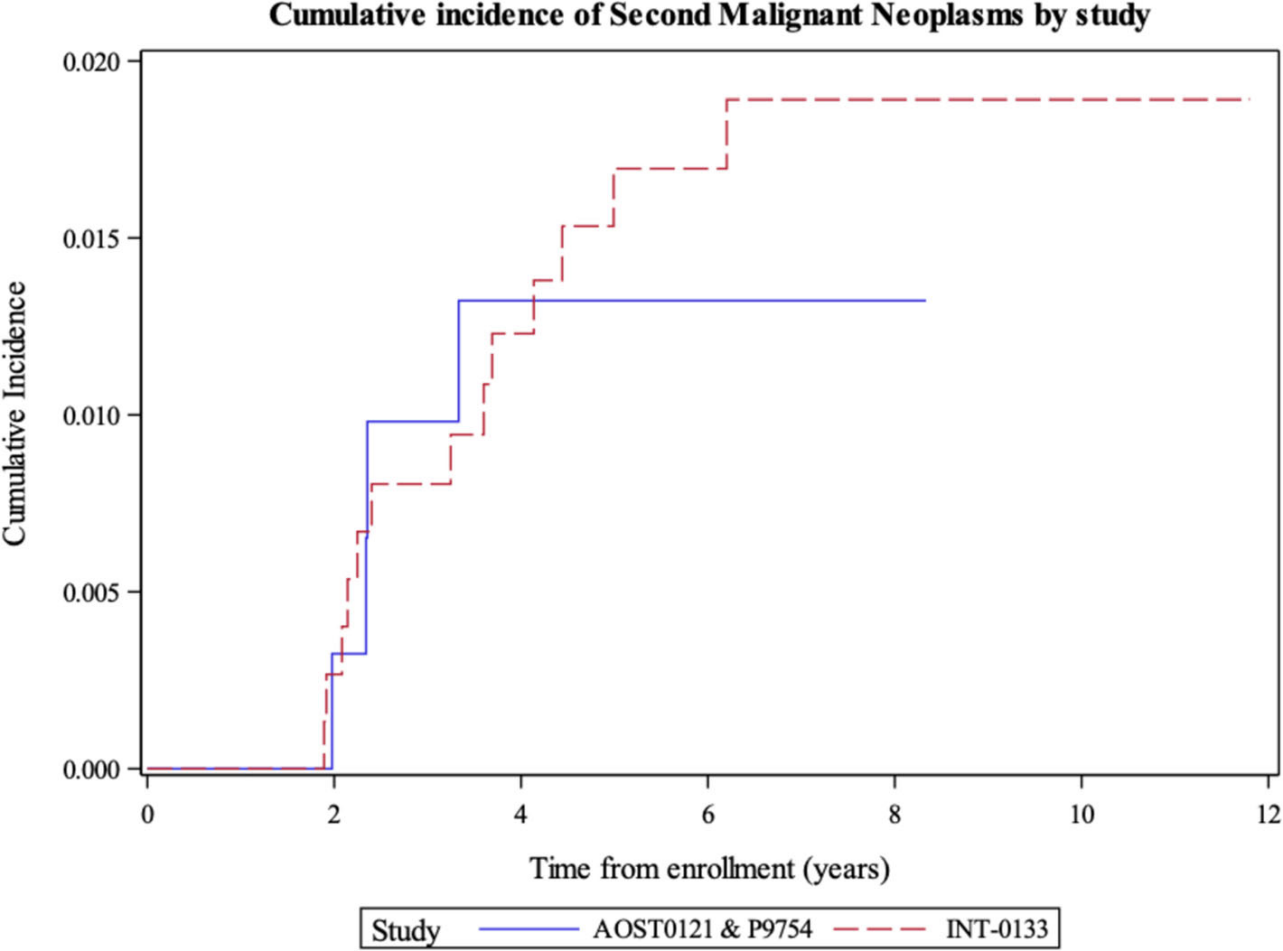
5-year Cumulative Incidence of Second Malignant Neoplasms in The Clinical Trial INT-0133 versus COG P9754 and COG AOST0121

There were five patients with SMNs in P9754 and AOST0121 combined: 3 patients with acute myelogenous leukemia, 1 patient with myelodysplastic syndrome, and 1 patient with juvenile myelomonocytic leukemia. Four were first events and one occurred after relapse of osteosarcoma. Three of the patients were enrolled on AOST0121 and as part of the protocol were scheduled to receive etoposide with ifosfamide. The other two patients were enrolled on P9754, Pilot 2, and were not scheduled to receive etoposide and ifosfamide. The five-year cumulative incidence of SMNs was 1.3% (95% CI, 0.44 to 3.2%). This was similar to the 1.7% five-year SMNs cumulative incidence in INT-0133 (95% CI, 0.93 to 2.9%) (*P* = 0.65), which did not use dexrazoxane (Figure 4).

## Discussion

We reviewed data on 315 patients enrolled on two COG OS trials (P9754 and AOST0121), all of whom received higher cumulative dose doxorubicin with dexrazoxane cardioprotection. None of the 315 patients enrolled on either study had reported clinical cardiac toxicity after treatment with 450–600 mg/m^2^ of doxorubicin as per NCI-CTCAE (Common Terminology Criteria for Adverse Events) version 2, with or without trastuzumab and concomitant dexrazoxane [12, 13]. We also reviewed detailed cardiac function data of in 81 patients enrolled on the two trials who had such data available. Many patients, especially girls, had echocardiographic abnormalities or elevated concentrations of NT-proBNP after treatment. Left ventricular end-diastolic septal thickness and LV mass were significantly smaller for BSA than normal for both sexes. These echocardiographic changes persisted: one-third of children evaluated > 81 days after the METT had significantly smaller LV posterior wall thicknesses for BSA than normal controls. Girls also had a significantly smaller LV dimension *Z-*score which indicates greater susceptibility to doxorubicin cardiotoxicity [7, 15, 28, 29].

Anthracyclines target topoisomerase IIβ to cause DNA double-strand breaks, impairing transcription and translation, and they also couple with iron to generate reactive oxygen species. These species, in turn, cause an irreversible cardiomyocytic mitochondriopathy [8, 9]. Dexrazoxane chelates myocardial iron, preventing it from coupling with anthracyclines, thus preventing the mitochondriopathy [19].

Dexrazoxane is an effective cardioprotectant in children with acute lymphoblastic leukemia (ALL) [25, 30–32]. In an early use of dexrazoxane in children with sarcomas, 38 children were randomly assigned to receive doxorubicin with or without dexrazoxane [33]. Those children receiving dexrazoxane were less likely to have subclinical cardiotoxicity and had smaller declines in LV ejection fraction [33]. In the current cohort of patients with OS, none experienced clinical heart failure [12, 13], and none had any marked changes in LV fractional shortening *Z-*scores (Table 5 *P* = 0.30), or in LV end diastolic septal thickness *Z-*scores (Table 5 *P* < 0.01) despite treatment with trastuzumab and/or with cumulative doxorubicin doses up to 600 mg/m^2^. Although our study was not randomized, we believe the minimal cardiac changes noted are likely attributable to the significant cardioprotective benefit of dexrazoxane.

In studies of women with breast cancer, trastuzumab plus doxorubicin had rates of clinical cardiotoxicity as high as 27% [34]. Trastuzumab alone had a relative risk of 5.11 for severe heart failure in a meta-analysis of women with breast cancer [35]. A retrospective study of women with HER2-positive breast cancer who received doxorubicin and trastuzumab showed that the 25% who received dexrazoxane had significantly fewer cardiac events [36]. Our study supports this impression that dexrazoxane may mitigate the cardiotoxicity of doxorubicin given with trastuzumab.

In the > 200 children with ALL randomly assigned to receive doxorubicin alone or with dexrazoxane, cTnT and NT-proBNP concentrations increased significantly in children who received doxorubicin alone [31]. These increases were related to abnormal echocardiographic findings 4 years later [25]. In the OS studies reported here, all patients received dexrazoxane, and none of the evaluable children and adolescents had elevated concentrations of cTnT. The NT-proBNP concentrations overall did not reach levels of concern for heart failure risk in either study, with only one girl having a value in the range of heart failure risk (Figures 2 and 3). In P9754, NT-proBNP concentrations were significantly higher in girls than in boys at the end of therapy and remained high but never indicated heart failure (Figure 2). In AOST0121 we had few NT-proBNP measurements available for analysis. At the end of therapy some measurements were elevated for boys, which decreased with time. The findings in boys are consistent with the acute cardiotoxicity profile of trastuzumab, as the cardiotoxicity is often reversible once it is stopped [34, 35, 37, 38]. In the girls NT-proBNP slightly increased yet overall the concentration did not reach cardiomyopathy risk (Fig. 3).

Our finding that girls had more progressive abnormalities of LV structure (decreased LV wall thickness *Z*-scores, LV mass *Z*-scores and LV end-diastolic dimension *Z-*scores) than boys indicates that doxorubicin-treated girls have hearts that are disproportionately small for body size, increasing their ventricular stress as indicated by a statistically significant increase in NT-proBNP concentration. These results indicate that the dexrazoxane’s cardioprotection was incomplete for girls as reflected by the gender difference correlating with the smaller and more vulnerable hearts of girls. Other studies have also reported that female sex is an independent risk factor for late cardiac effects [7, 15, 28].

The 5-year cumulative incidence of SMNs in P9754 and AOST0121 combined was similar to historical controls from the INT-0133 study (Fig. 4). Doxorubicin itself is shown to increase SMN risk, as highlighted in a review of > 6000 childhood solid tumor survivors, which found a doxorubicin-dose-dependent increased SMN risk [39].

Some clinicians have hesitated to use dexrazoxane in children and adolescents with cancer because of a reported possible association between dexrazoxane and an increased risk of SMNs in patients with Hodgkin’s lymphoma [10]. In those studies, uniquely, three topoisomerase inhibitors (etoposide, doxorubicin, and dexrazoxane) were used simultaneously. Chow et al. reviewed these same Hodgkin’s lymphoma studies (POG 9425 and 9426), and the POG 9404 T-cell lymphoblastic lymphoma/leukemia study. With longer follow-up there was no increase in secondary AML/MDS attributable to dexrazoxane use [40]. In a multicenter study of 205 patients with high-risk ALL, half of whom received dexrazoxane with doxorubicin chemotherapy, dexrazoxane did not compromise the efficacy of doxorubicin [31, 32, 40], and there was no increase in the cumulative incidence of SMNs associated with dexrazoxane after a median follow-up of 6.2 years [41]. Similar findings were seen in other studies [30, 42], one of which examined 15,532 anthracycline-treated pediatric cancer patients, of whom 1406 received dexrazoxane, and found no increased risk of secondary AML [42]. In yet another set of sequential childhood protocols, among 553 high-risk ALL patients treated with dexrazoxane the only SMN was a single case of AML. The overall 5-year confidence interval of SMNs for patients was lower than the range in most historical studies, indicating SMNs were rare [43]. This updated SMN data led the European Medicines Agency (EMA) to withdraw its prohibition of dexrazoxane use in children aged 0–18 years. It now allows the use of dexrazoxane in Europe for children from the start of anthracycline chemotherapy if the planned cumulative dose is over 300 mg/m^2^. Their review also did not show evidence of dexrazoxane interference with chemotherapy, and led to removal of a safety warning for early death associated with dexrazoxane [44].

Our study was prospective, and all echocardiograms were centrally reviewed. However, the studies were not randomized so there were no comparison arms, and the numbers of children and adolescents with available data after the expected completion of treatment were small (echo data is missing in 74%, troponin in 78% and BNP in 81% of patients). Also, as the follow up period was short it is possible patients may have developed cardiotoxicity later in follow up. The substantial amount of missing data could have biased our results. Nonetheless, the absence of cardiotoxicity in children and adolescents receiving high-dose anthracycline therapy supports the conclusion that dexrazoxane is cardioprotective. There was no suggestion of an increased incidence in SMN.

## Conclusions

Our study adds to the literature derived from randomized trials that show evidence of dexrazoxane cardioprotection [30, 32, 33, 45]. As the number of childhood cancer survivors increases [46] the use of dexrazoxane may reduce the total cumulative burden for this vulnerable and medically complex population. Initial data from the ongoing COG ALTE11C2 protocol has demonstrated that with 16 years follow-up dexrazoxane is associated with statistically significant long-term cardioprotection, as evidenced by better LV function (fractional shortening) and lower levels of brain natriuretic peptides in multivariate analyses, when compared with anthracycline-treated patients who did not receive dexrazoxane [45]. Further, pharmacoeconomic analyses have indicated that the balance of additional costs driven by dexrazoxane compared with savings from a reduction in cardiology-associated costs and hospitalizations favored the use of dexrazoxane, a point that is important as we strive to reduce survivors’ chronic health condition burden [4, 47].

Our data support the conclusion that dexrazoxane should be recommended for use in all children and adolescents with OS from the initiation of doxorubicin therapy, particularly in girls, who exhibit more cardiotoxicity than boys at equal cumulative doses. This is consistent with the new EMA indications and also takes into account that irreversible cardiomyocyte injury occurs from the first anthracycline dose, regeneration of cardiomyocytes is limited, drug therapy can only partially restore normal cardiac function, and the incidence of cardiac damage will increase with the duration of survival of pediatric cancer patients [4, 48].

## Data Availability

The data that support the findings of this study are available from Children’s Oncology Group but are not publicly available. Data are however available from the authors upon reasonable request and with permission of the Children’s Oncology Group.

## Abbreviations

ALL: Acute lymphoblastic leukemia
AML: Acute myeloid leukemia
BSA: Body-surface area
COG: Children’s Oncology Group
cTnT: Serum cardiac troponin T concentrations
EMA: European Medicines Agency
HER2: Human epidermal growth factor receptor 2
IE: Ifosfamide and etoposide
LV: Left ventricular
MAP: Methotrexate, doxorubicin, cisplatin
MDS: Myelodysplastic syndrome
METT: Minimum expected treatment time
NT-proBNP: Serum N-terminal pro-brain natriuretic peptide concentration
OS: Osteosarcoma
SMN: Second malignant neoplasm
